# Neuronal Differentiation of Human Glioma Cells Induced by Parthenolide Under In Vitro Conditions

**DOI:** 10.3390/biomedicines12112543

**Published:** 2024-11-07

**Authors:** Zhaoqi Tang, Chang Cao, Weiwei Tang, Yanrong Ye, Zhenhui Chen, Yun Shen

**Affiliations:** 1 Department of Pharmacy, Zhongshan Hospital (Xiamen), Fudan University, Xiamen 361015, China; 2 Xiamen Clinical Research Center for Cancer Therapy, Xiamen 361015, China; 3 Department of Medical Oncology, The First Affiliated Hospital of Xiamen University, School of Medicine, Xiamen University, Xiamen 361003, China; 4 Department of Medical Oncology, The First Affiliated Hospital of Xiamen University, School of Clinical Medicine, Fujian Medical University, Xiamen 361003, China; 5 Xiamen Key Laboratory of Antitumor Drug Transformation Research, The First Affiliated Hospital of Xiamen University, Xiamen 361003, China; 6 Department of Pharmacy, Zhongshan Hospital, Fudan University, Shanghai 200032, China

**Keywords:** parthenolide, glioma, differentiation, neuron, proliferation, histone deacetylase

## Abstract

**Objective:** Previous drug repositioning studies have suggested that parthenolide may be a differentiation-inducing agent for glioma cells. This study aimed to experimentally verify the neuronal differentiation-inducing effects and proliferative impact of parthenolide on human glioma cells and explore its potential mechanisms. **Methods:** HE staining was used to observe the morphological changes in human glioma cell lines U87 and A172 induced by parthenolide. Immunocytochemistry was conducted to detect the expression of differentiation markers. The Ki-67 detection and CCK-8 assay were used to assess the effects of parthenolide on cell proliferation. The sphere formation assay was conducted to evaluate the self-renewal. Glioma stem cells (GSCs) derived from U87 cells were utilized to assess the ability of parthenolide to induce differentiation in GSCs. Western blot was used to detect the expression of histone deacetylase 1 (HDAC1). Bioinformatics analysis based on the CGGA database was conducted to evaluate the role of HDAC1 in glioma. **Results:** Parthenolide (4 μM) altered the morphology of U87 and A172 cells, as elongated cell projections were observed. Parthenolide induced glioma cells to express neuronal markers NeuN, MAP2, SYP, and NEFL, but not astrocyte or oligodendrocyte markers. Parthenolide significantly inhibited proliferation and self-renewal in glioma cells. Similar effects were observed in U87 GSCs. Furthermore, parthenolide downregulated HDAC1 expression in glioma cells, and the bioinformatics analysis revealed a potential relationship between neuronal characteristics and low expression of HDAC1 in glioma. **Conclusion:** Parthenolide induced neuronal differentiation and inhibited the cell proliferation in human glioma cells, which might be associated with the inhibition of HDAC1.

## 1. Introduction

Glioma is the most common primary malignant brain tumor in adults. Glioblastoma multiforme (GBM), the most prevalent type of glioma, is a highly malignant grade 4 diffuse glioma. The median survival for untreated patients is 3–4.5 months, and maximal treatment with debulking surgery followed by chemoradiotherapy only increases the median survival to 15–16 months, with a 2-year survival rate of 27–31% and a 5-year survival rate of 7–10% [1]. Therefore, more effective treatment strategies for glioma are needed. Owing to the infiltrative nature of glioma, complete surgical resection is difficult. Residual glioma stem cells (GSCs) are characterized by self-renewal and heterogeneity, as well as resistance to chemotherapeutic agents such as temozolomide, leading to tumor recurrence, which is a significant cause of treatment failure [2]. The induction of glioma cell differentiation and attenuation of stemness in GSCs may play crucial roles in the treatment of glioma and prevention of postoperative recurrence. Some scholars have researched the induction of glioma cell differentiation. For example, cyclic adenosine monophosphate (cAMP) activators can induce the neuronal differentiation of GSCs [3], the upregulation of the expression of the transcription factors nuclear factor 1 A (NFIA) and nuclear factor 1 B (NFIB) can induce the differentiation of GBM cells into astrocytes [4], and the downregulation of zinc finger protein 117 (ZNF117) expression can induce the differentiation of GSCs into oligodendrocytes [5]. These differentiation induction treatments significantly inhibited the proliferation of glioma cells, indicating that differentiation induction is a promising therapeutic strategy for glioma.

Parthenolide is a natural sesquiterpene lactone produced as secondary metabolite by plants of the Asteraceae/Compositae (daisies) and Magnoliaceae family (magnolias), and is the main biologically active ingredient of feverfew. It exhibits promising anti-inflammatory and anti-tumor properties [6]. Parthenolide has been shown to inhibit nuclear transcription factor-κB (NF-κB) signaling and other prosurvival signaling pathways, induce apoptosis, and reduce a subpopulation of cancer stem-like cells in several cancers [7]. In our previous study of drug repositioning via the Connectivity Map database, we reported that parthenolide may have a differentiation-inducing effect on glioma cells [8], but there are currently no relevant experimental research reports on this topic. This study aimed to experimentally verify the differentiation-inducing and proliferation-inhibiting effects of parthenolide on glioma cells and explore its potential mechanism.

## 2. Materials and Methods

### 2.1. Cell Culture and Reagents

Human glioma cell lines U87 and A172 were purchased from the Cell Bank of Chinese Academy of Sciences and cultured in DMEM (HyClone, Logan, UT, USA) supplemented with 10% fetal bovine serum (ExCell, Shanghai, China) and 1% penicillin/streptomycin (BasalMedia, Shanghai, China). Cells were maintained at 37 °C in a humidified atmosphere containing 5% CO_2_. The drug treatment group was cultured with complete culture medium containing parthenolide (Macklin, Shanghai, China), while the control group was cultured with complete culture medium containing DMSO solvent.

U87 GSCs were isolated from the U87 cell line. U87 cells were cultured in ultra-low attachment culture dishes using DMEM/F12 (HyClone, USA) medium supplemented with 2% B27 supplement (iCell, Shanghai, China), 20 ng/mL epidermal growth factor (EGF) (Novoprotein, Beijing, China), and 20 ng/mL basic fibroblast growth factor (bFGF) (Novoprotein, China). After 5 days, the cell spheres were collected using a 70 mm cell strainer. The cell spheres were digested and dispersed with trypsin and then cultured in the same manner. The cell spheres collected for the third time were used as GSCs. For differentiation, poly-L-Lysine-coated cell crawling slides were used.

### 2.2. Hematoxylin-Eosin (HE) Staining

Cells were cultured in 6-well plates and subsequently treated with parthenolide for 3 days. Then, cells were fixed using 4% paraformaldehyde for 10 min and stained with hematoxylin (Biosharp, Hefei, China) for 5 min and eosin (Biosharp, China) for 10 s. The images were captured via an inverted microscope (Nikon, Tokyo, Japan).

### 2.3. Immunocytochemistry

Cells were cultured in 6-well plates and subsequently treated with parthenolide (4 μM) for 3 days. Then, cells were fixed using 4% paraformaldehyde for 10 min, permeabilized with methanol at 4 °C for 10 min, blocked with 5% bull serum albumin for 1 h, then incubated with primary antibodies at 4 °C overnight. After that, cells were incubated with secondary antibody at room temperature for 1 h, followed by staining with DAPI for 10 min, and finally mounted with antifade mounting medium. The images were captured via an inverted microscope (Nikon, Tokyo, Japan). The primary antibodies used included the following: microtubule-associated protein 2 (MAP2) antibody (Abcam, Cambridge, UK), neuronal nuclei antigen (NeuN) antibody (Abcam, Cambridge, UK), synaptophysin (SYP) antibody (Beyotime, Shanghai, China), neurofilament light chain (NEFL) antibody (Proteintech, Shanghai, China), glial fibrillary acidic protein (GFAP) antibody (CST, Peachtree City, GA, USA), S100 calcium binding protein B (S100B) antibody (ABclonal, Wuhan, China), oligodendrocyte transcription factor 2 (Olig2) antibody (CST, USA), myelin basic protein (MBP) antibody (Novus, Chesterfield, MO, USA), and Ki-67 antibody (CST, USA).

### 2.4. Ki-67 Positive Rate Calculation

For each sample, five fields of view were randomly selected and photographed under 200× magnification. The Image-Pro Plus 6.0 software was used to count Ki-67 stained cells and DAPI-stained cells in the same field of view. The experiment was repeated three times, and the positive rate of each field of view was calculated and averaged.

### 2.5. Cell Viability Assay

Cells were cultured in 96-well plates and subsequently treated with parthenolide (4 μM) for 3 days. Then, 10 μL of CCK-8 (Glpbio, Montclair, CA, USA) reagent was added to each well of the plates, and the cells were incubated at 37 °C for 1 h. The optical density at 450 nm was read with a microplate reader (Thermo Scientific, Waltham, MA, USA). Four replicate wells were set for each group. The experiment was repeated three times.

### 2.6. Sphere Formation Assay

Cells were cultured in ultra-low attachment 12-well plates with DMEM/F12 medium supplemented with 2% B27 supplement, 20 ng/mL EGF, and 20 ng/mL bFGF for 5 days. For each sample, three random fields of view were photographed under 100× magnification. The diameters of the neurospheres were measured using ImageJ 1.51j8 software. The total number of neurospheres with diameters greater than 75 μm in the three fields of view was also calculated. The experiment was repeated three times.

### 2.7. Western Blot

Cells were cultured in 6-well plates and subsequently treated with parthenolide (4 μM) for 3 days. Protein samples were separated by SDS-PAGE and then transferred to an NC membrane. The membrane was blocked with 5% BSA on a shaker at room temperature for 1 h. The antibody was diluted with blocking solution, and the membrane was incubated with primary antibody overnight at 4 °C. After that, the membrane was washed with PBST three times, each for 10 min, and then incubated with the secondary antibody at room temperature in the dark for 1 h. This was followed by washing the membrane with PBST three times, each for 10 min. After adding the ECL agent, the membrane was imaged. The images were analyzed via ImageJ software.

### 2.8. Chinese Glioma Genome Atlas (CGGA) Analysis

The mRNAse_325 dataset was downloaded from the CGGA database (http://www.cgga.org.cn/, accessed on 28 September 2023). The samples were grouped according to the quartile of target gene expression, and those above the quartile were divided into the high group and those below the quartile were divided into the low group. Genes with |Log2FC| values greater than 3 and *p* values less than 0.05 were considered as the differentially expressed genes (DEGs) between the high and low groups. The differences in gene expression, tumor grade, and survival between groups were analyzed.

### 2.9. Enrichment Analysis

The gene symbols were uploaded to the Search Tool for Retrieval for Interaction Gene/Proteins (STRING) database (https://cn.string-db.org/, accessed on 7 October 2023) for protein–protein interaction (PPI) network creation and to the Database for Annotation, Visualization, and Integrated Discovery (DAVID, https://david.ncifcrf.gov/, accessed on 7 October 2023) for Gene Ontology (GO) enrichment analysis.

### 2.10. Statistical Analysis

Statistical analysis was performed using SPSS (International Business Machines Corporation, Armonk, NY, USA) 16.0 software. A *t* test was used to compare the mean values between groups. The log-rank test was used to compare the survival curves. The chi-square test was used to compare tumor grade compositions between groups. A *p* value less than 0.05 was considered statistically significant.

## 3. Results

### 3.1. Parthenolide Induced Morphological Changes in Glioma Cells

**Figure 1:**
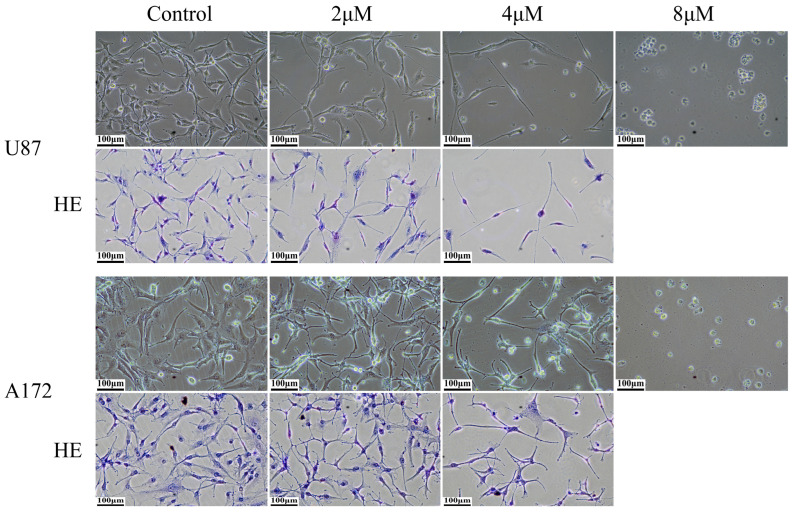
Effects of parthenolide on the morphology of glioma cells (200×), scale bar = 100 μm. Elongated cell projections appeared after parthenolide (4 μM) treatment for 3 days.

Cell microscopy images showed the cell morphology, and HE staining was performed to enhance the visualization of cell morphology. Cell microscopy images and HE staining results revealed that parthenolide had a relatively minor effect on the morphology of U87 and A172 cells at a concentration of 2 μM. At a concentration of 4 μM, some dead cells were observed, and surviving cells exhibited some morphological changes, such as the development of elongated cell projections. At a concentration of 8 μM, all the cells died (Figure 1). Therefore, a concentration of 4 μM was chosen for subsequent experiments.

### 3.2. Parthenolide Induced the Expression of Neuronal Markers in Glioma Cells

**Figure 2:**
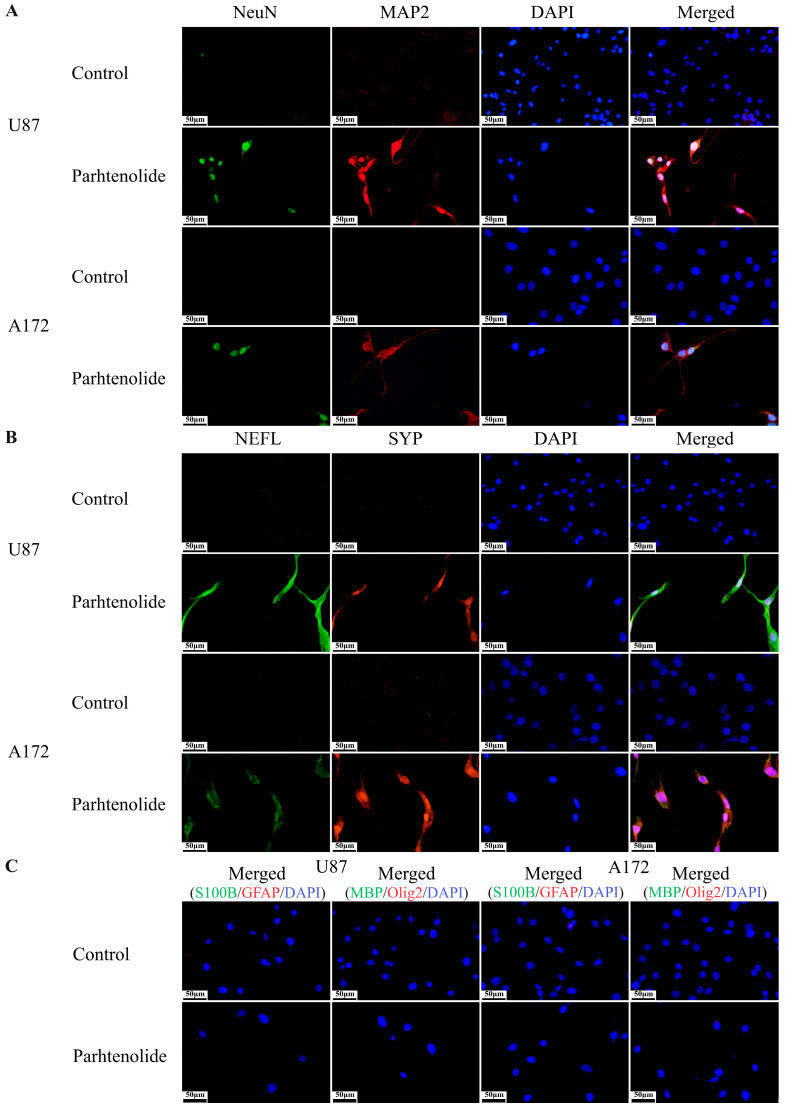
Effects of parthenolide on the expression of differentiation markers in glioma cells (400×), scale bar = 50 μm. Cells expressed neuronal markers, but not astrocyte or oligodendrocyte markers, after parthenolide (4 μM) treatment for 3 days. (**A**) Expression of neuronal markers NeuN and MAP2; (**B**) expression of neuronal markers SYP and NEFL; (**C**) expression of astrocyte and oligodendrocyte markers.

The immunocytochemistry results showed that the expression of neuronal markers NeuN, MAP2, SYP, and NEFL was detected in the parthenolide treatment group, but not in the control group (Figure 2A,B). Additionally, no expression of astrocyte markers GFAP and S100B or oligodendrocyte markers MBP and Olig2 was detected after parthenolide treatment (Figure 2C).

### 3.3. Parthenolide Inhibited the Proliferation of Glioma Cells

**Figure 3:**
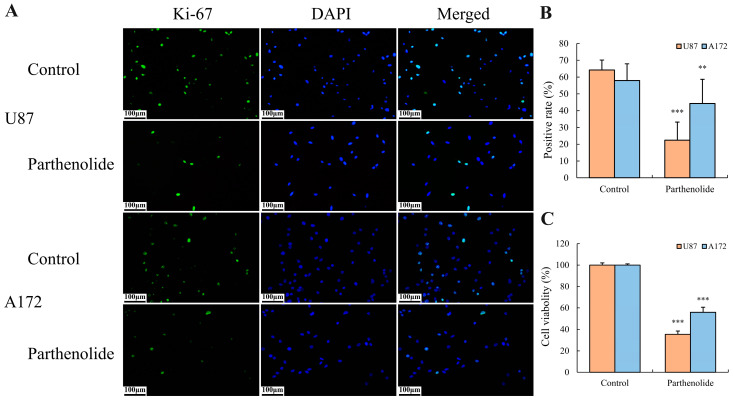
Effects of parthenolide on the proliferation of glioma cells. Parthenolide (4 μM) significantly inhibited the proliferation of U87 and A172 cells. (**A**) Immunocytochemistry detection of Ki-67 (200×), scale bar = 100 μm; (**B**) positive rates of Ki-67 (*n* = 3); (**C**) cell viability detected by the CCK-8 assay (*n* = 3). ** *p* < 0.01 vs. control group, *** *p* < 0.001 vs. control group.

The immunocytochemistry of Ki-67 showed that the Ki-67 positive rates of U87 and A172 cells in the parthenolide treatment groups were significantly lower than those in the corresponding groups (Figure 3A,B). Additionally, the results of the CCK-8 assay indicated that the viability of U87 and A172 cells significantly decreased after parthenolide treatment (Figure 3C). These findings suggested that parthenolide significantly inhibited the proliferation of glioma cells.

### 3.4. Parthenolide Inhibited the Self-Renewal of Glioma Cells

**Figure 4:**
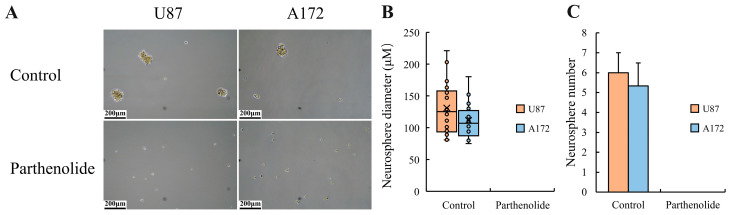
Effects of parthenolide on the self-renewal of glioma cells (*n* = 3). Parthenolide (4 μM) significantly inhibited the sphere formation. (**A**) Microscopy images of neurosphere formation (100×), scale bar = 200 μm; (**B**) neurosphere diameters; (**C**) neurosphere numbers.

The results of the sphere formation assay show that, in the control groups of U87 and A172 cells, clear neurospheres were formed, whereas in the parthenolide groups, no neurospheres with a diameter greater than 75 μm were observed (Figure 4), indicating that parthenolide significantly inhibits the self-renewal of glioma cells.

### 3.5. Parthenolide Induced the Differentiation and Proliferation Inhibition of GSCs

**Figure 5:**
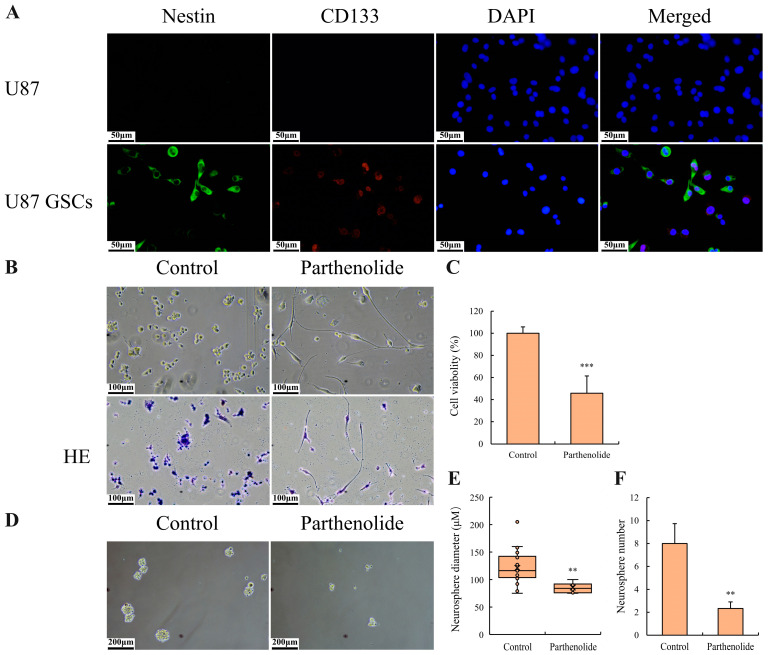
Effects of parthenolide on the differentiation and proliferation of U87 GSCs. Parthenolide (4 μM) induced differentiation and inhibited the cell proliferation in U87 GSCs. (**A**) NSC markers detected by immunocytochemistry (400×), scale bar = 50 μm; (**B**) morphological changes induced by parthenolide (200×), scale bar = 100 μm; (**C**) cell viability detected by a CCK-8 assay (*n* = 3); (**D**) microscopy images of neurosphere formation (100×), scale bar = 200 μm; (**E**) neurosphere diameters (*n* = 3); (**F**) neurosphere numbers (*n* = 3). ** *p* < 0.01 vs. control group, *** *p* < 0.001 vs. control group.

The immunocytochemistry results showed that NSC markers nestin and CD133 were expressed in U87 GSCs (Figure 5A). Under microscopic observation, U87 GSCs appeared approximately round, and after parthenolide treatment, they developed neuron-like elongations (Figure 5B). Additionally, cell proliferation was inhibited as well (Figure 5C). Sphere formation assays demonstrated that, compared with the control group, the parthenolide group presented significantly fewer and smaller neurospheres, indicating that parthenolide attenuated the stemness of U87 GSCs.

### 3.6. Inhibition of Histone Deacetylase 1 (HDAC1) Is a Potential Mechanism

It is reported that HDAC inhibitors can induce neuron-like differentiation in glioma cells [9,10]. Additionally, parthenolide was found to be a HDAC1 inhibitor [6]. Therefore, we hypothesized that the differentiation-inducing effect exhibited by parthenolide may be associated with HDAC1. To further investigate, we explored the potential role of HDAC1 in glioma.

**Figure 6:**
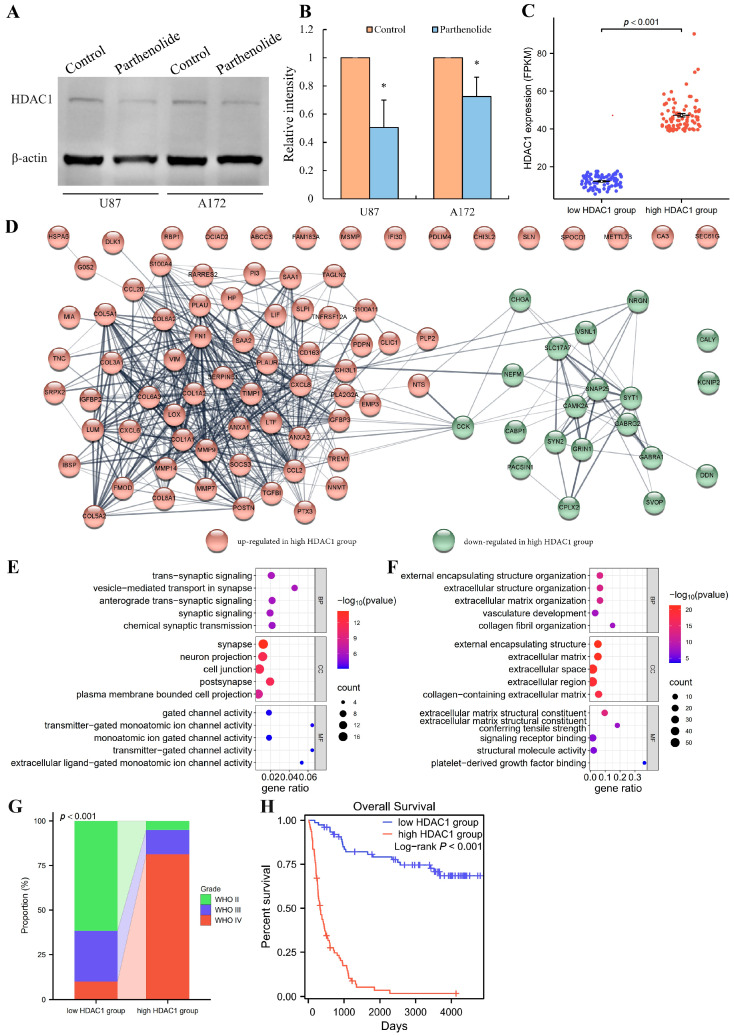
Potential role of HDAC1 in neuronal differentiation induced by parthenolide. (**A**) Western blot bands showing the effects of parthenolide on the expression of HDAC1 in glioma cells; (**B**) relative expression level of HDAC1 based on Western blot bands (*n* = 3), * *p* < 0.5 vs. control group; (**C**) expression level of HDAC1 in the high- and low-HDAC1 groups of glioma samples in the CGGA database; (**D**) PPI network of the DEGs between the high and low groups; (**E**) GO enrichment analysis of the upregulated genes in the high-HDAC1 group; (**F**) GO enrichment analysis of the downregulated genes in the high-HDAC1 group; (**G**) tumor grade composition of the two groups; (**H**) comparison of survival between the two groups, *n* (low-HDAC1 group) = 81, *n* (high-HDAC1 group) = 81.

The Western blot results demonstrated that parthenolide significantly downregulated the expression of HDAC1 in U87 and A172 cells (Figure 6A,B). Differences in gene expression between glioma samples with high and low HDAC1 expression were analyzed via the CGGA database. There was a statistically significant difference in HDAC1 expression between the high and low expression groups (Figure 6C). The PPI network revealed that the upregulated and downregulated genes in the high-HDAC1 group could be roughly divided into two clusters (Figure 6D), so enrichment analysis was performed on these genes separately. For the upregulated genes, the biological processes (BP), cellular components (CC), and molecular functions (MF) in which the genes were enriched were mainly related to the extracellular matrix (ECM) (Figure 6E). For the downregulated genes, the enrichment terms were mainly related to synapses (Figure 6F). Moreover, the proportion of tumor grade was significantly different between the two groups, with the high-HDAC1 group having a higher proportion of WHO grade 4 (GBM) (80.25%) than the low-HDAC1 group did (9.88%) (Figure 6G). Overall survival was lower in the high-HDAC1 group than in the low-HDAC1 group (Figure 6H).

## 4. Discussion

This study investigated the differentiation-inducing effects of parthenolide on human glioma cells and its impact on cell proliferation, as well as the potential mechanisms. The results showed that treatment with a 4 μM concentration of parthenolide for three days significantly altered the morphology of U87 and A172 cells; induced the expression of neuronal markers NeuN, MAP2, SYP, and NEFL; and inhibited their proliferation and self-renewal. Furthermore, parthenolide also suppressed the expression of HDAC1 in glioma cells, and bioinformatics analysis suggested that this effect may be related to its ability to induce neuronal differentiation.

Due to the lack of proliferative capacity in terminally differentiated neurons, some studies have focused on inducing the differentiation of glioma cells into neurons in recent years. For example, Chen et al. [3] demonstrated that cAMP activators induced the neuronal differentiation of GSCs and inhibited glioma growth in mouse xenograft models. Liu et al. [9] reported that the HDAC inhibitor MS-275, in combination with cAMP activators, could induce U87 cells and GSCs to differentiate into neuron-like cells, and these neuronal transformations significantly inhibited the proliferation of glioma cells. Liu et al. [10] reported that the HDAC inhibitor sodium butyrate induced U87 cells to differentiate into cholinergic neurons and inhibited their proliferation. Our study revealed that parthenolide treatment changed the morphology of U87 and A172 cells and induced the expression of neuronal markers without the expression of astrocyte or oligodendrocyte markers, indicating that parthenolide induced U87 and A172 cells to differentiate into neuron-like cells. Further research revealed that after parthenolide treatment, in addition to a significant decrease in overall cell viability, the Ki-67 positivity rates of surviving cells also decreased significantly, indicating that the induction of differentiation by parthenolide attenuated the proliferative capacity of glioma cells. Inducing neuronal-like differentiation of glioma cells and thereby inhibiting their proliferation is involved in the anti-glioma mechanism of parthenolide.

The clinical course of GBM is characterized by almost inevitable recurrence within the first year of treatment and significant resistance to chemotherapy and radiotherapy. Low proliferative activity, treatment resistance, and associations with tumor recurrence are typical characteristics of GSCs [11]. To investigate whether parthenolide has a differentiation-inducing effect on GSCs, U87 GSC-like cells were utilized in this study. The results indicated that parthenolide induced the differentiation of GSC-like cells and inhibited their proliferation and self-renewal, suggesting that parthenolide has the potential to prevent glioma recurrence.

Previous studies have shown that the molecular mechanisms underlying the antiglioma effects of parthenolide are related mainly to the inhibition of Akt and NF-κB. Additionally, it reduces temozolomide resistance by downregulating O-6-Methylguanine-DNA Methyltransferase (MGMT) expression [12,13]. In other cancers, the anticancer mechanism of parthenolide is believed to be mainly related to the inhibition of NF-κB, and other mechanisms include the inhibition of mouse double minute 2 homolog (MDM2), HDAC1, transcription protein 3 (STAT3), and glutathione (GSH) [7]. Since HDAC inhibitors have been found to induce neuronal-like differentiation in glioma cells [9,10], and there are currently no reports on such effects related to the drug targets of parthenolide except HDAC1, we focused our attention on HDAC1. HDACs are important chromatin-modifying enzymes that catalyze the deacetylation of lysine residues in histone. Deacetylated histones maintain their positive charge and remain tightly bound to negatively charged DNA, thus limiting the accessibility of transcription factors and suppressing gene expression. HDAC1 belongs to class I HDACs [14]. Most HDAC inhibitors are not specific, while parthenolide selectively depletes HDAC1 in micromolar concentrations through ataxia telangiectasia mutated (ATM) activation, leaving other HDACs unaffected [15]. This study confirms the inhibitory effect of parthenolide on HDAC1 in glioma cells. Recent studies on HDAC1 in glioma have shown that HDAC1 is a necessary class I HDAC in GSCs that maintains the malignant phenotype of GSCs and promotes the progression of GBM [16,17]. Furthermore, HDAC1 is an important component of the RE-1 silencing transcription factor (REST) complex. REST is an inhibitory transcription factor that targets multiple neuronal genes. NSCs express high levels of REST, which inhibits the expression of neuronal genes and helps maintain the stem cell state, but its expression is downregulated during neuronal development, allowing for extensive transcription of neuron-specific genes [18]. These findings suggest a potential association of HDAC1 with neuronal differentiation. Bioinformatics analysis in this study indicated that high expression of HDAC1 in glioma was associated with the extracellular matrix (ECM) and poor prognosis. The ECM is a non-cellular component of tissues that provides biochemical and structural support to cells and has been shown to guide cell migration and proliferation [19]. In glioma cells, the synthesis of the ECM is upregulated, thereby inducing the expression of invasion-related molecules [20]. After surgery, residual glioma cells after surgery actively create and remodel a more abundant, denser, and stiffer ECM, promoting glioma recurrence and progression through stemness or aggression [21]. These findings suggest that high expression of HDAC1 may be associated with the high invasiveness and recurrence of glioma. Samples with low HDAC1 expression were associated with more neuronal features and better prognosis, indicating that inhibiting HDAC1 may be associated with the neuronal-like differentiation of glioma cells induced by parthenolide.

## 5. Conclusions

In summary, this study suggested that parthenolide induced the differentiation of glioma cells into neuron-like cells and inhibited cell proliferation. The inhibition of HDAC1 expression in glioma cells may potentially be associated with these effects, although further investigation is needed to confirm the underlying mechanism. We will continue to explore the role of HDAC1 in the neuronal differentiation induced by parthenolide and the molecular mechanisms involved in this process in subsequent studies. This study confirmed the anti-glioma effect of parthenolide and supplemented the understanding of its anti-glioma mechanism, which has the potential to inform future clinical trials and the development of new therapeutic strategies for glioma patients. This study also verified the reliability of using the Connectivity Map database for drug repositioning.

## Data Availability

The original data presented in the study are openly available in FigShare at 10.6084/m9.figshare.27088660.
